# Evaluating the Cause of Death in Obese Individuals: A Ten-Year Medical Autopsy Study

**DOI:** 10.1155/2015/695374

**Published:** 2015-01-14

**Authors:** Jad Saab, Steven P. Salvatore

**Affiliations:** NewYork-Presbyterian Hospital, Weill Cornell Medical College, Department of Pathology and Laboratory Medicine, 525 East 68th Street, New York, NY 10065, USA

## Abstract

*Background*. Obesity is a growing public health problem associated with increased morbidity and rate of death. Postmortem examination is imperative to determine the cause of death, to detect clinically unsuspected disease entities, and consequently to determine the actual impact of obesity on patient mortality. *Methods*. A total of 849 adult autopsies were retrospectively reviewed. Obese (BMI ≥ 30 kg/m^2^) and nonobese patients were separately studied. The primary cause of death in each group was categorized into malignancy, infection, stroke, ischemic and nonischemic heart disease, pulmonary embolism, hemorrhage, and primary nonneoplastic diseases of different organ systems. *Results*. Of 849 autopsies, 32.3% were obese. The leading causes of death in the obese population were malignancy (31.4%), infection (25.9%), ischemic heart disease (12.8%), and pulmonary embolism (6.2%). Obese individuals were statistically more likely to die from pulmonary embolism and liver disease and less likely to die from neurologic diseases and nonischemic heart disease. *Conclusion*. Autopsies on obese individuals constitute a third of all adult medical autopsies in our center. Increased death rates in the obese due to pulmonary embolism and liver disease should receive special clinical attention. Autopsy findings in the obese population should contribute to overall premortem disease detection, prevention, and management.

## 1. Introduction

Obesity is a growing public health problem with multiple studies showing increased morbidity and rate of death with increasing body mass index (BMI) [1–5]. The World Health Organization (WHO) defines overweight as a BMI > 25 and ≤29.9 kg/m^2^ and obesity as a BMI ≥ 30 kg/m^2^ [6] and there appears to be a substantial long-term risk of becoming overweight or obese during adulthood. In the Framingham Offspring Study, Vasan et al. followed more than 4000 normal-weight white adults from 1971 to 2001. In a period of 40 years, 30% of the men and 19% of the women became overweight and 5% to 9% of the participants became obese. After 30 years of follow-up, more than half became overweight and approximately a third of the women and a fourth of the men became obese [7]. As of 2008, 31.8% of American adults over the age of 20 years were found to be obese [8], with annual obesity-related deaths ranging from 112,000 to 365,000 [9, 10].

The association between BMI and cause specific mortality was assessed in the Prospective Studies Collaboration analysis. The study found that, in individuals with a BMI between 25 and 50 kg/m^2^, every 5 kg/m^2^ increase in BMI was associated with an increased mortality from diabetes mellitus (DM), chronic kidney disease (CKD), ischemic heart disease (IHD), stroke, respiratory disease, and neoplasms [1]. However, these cases constitute only a subset of the deaths in the obese population.

In this study, we sought to review the postmortem findings in a large cohort of adult patients and compare the cause of death in obese and nonobese individuals. Postmortem examination is imperative to detect clinically unsuspected or undiagnosed disease entities, to determine the cause of death, and consequently to outline the actual impact of obesity on patient mortality.

## 2. Materials and Methods

All 849 consecutive adult autopsies from January 2003 until September 2013 performed at NewYork-Presbyterian Hospital/Weill Cornell Medical College were retrospectively reviewed.

Appropriate consent for the autopsy was signed by the spouse or legal next of kin as defined by New York State in accordance with the Decedent Estate Law (Public Health Law 4201 and article 205). Communication with the patient's clinician and clinical team was undertaken to determine if there were any specific concerns or questions that needed to be addressed. To be included in the study, the manner of death must be deemed “natural.” All other cases were referred to the New York City Office of Chief Medical Examiner. A thorough review of each patient's medical history including history of malignancy, hypertension (HTN), DM, elevated triglyceride level (HL), myocardial infarction (MI), and coronary artery revascularization procedures was performed. Laboratory tests and radiographic images were reviewed in all cases. Autopsies with restrictions, such as brain only, thorax only, and abdomen only and excluding head (or brain), were included only when a specific cause of death was identified. The Letulle technique was used during all postmortem examinations [12]. Bacterial cultures from blood and lung tissue were obtained using sterile techniques. Additional testing for viruses, fungi, and mycobacterial organisms was performed in select cases after review of the clinical chart. Photographs of the body as well as the external and cross-sectioned surfaces of the heart, lungs, kidneys, gastrointestinal tract, brain, and any unexpected findings were routinely taken. Reference ranges for weights and measurements of organs of adults were used [13].

Tissue was fixed in 10% neutral buffered formalin. Routine sections of the heart and major coronary arteries, lung lobes, kidneys, adrenals, pancreas, liver, bone marrow, spleen, thymus, thyroid, lymph nodes, breasts, ovaries, uterus, testes, prostate, and any lesional tissue were obtained. Significant coronary artery disease (CAD) was defined as >70% stenosis. Immunohistochemistry and special stains for microorganisms were performed as needed.

The patients were divided into obese and nonobese cohorts and studied separately. The obese cohort was further divided into WHO obesity classes I (BMI 30–34.9 kg/m^2^), II (BMI 35–39.9 kg/m^2^), and III (BMI ≥ 40 kg/m^2^) [14].

The primary cause of death was categorized into malignancy, infection, stroke, IHD, non-IHD, pulmonary embolism (PE), hemorrhage, and primary diseases of the kidney, liver, lung, skin, gastrointestinal tract, blood, and bone marrow and neuromuscular system. Patients with active malignancy who died of treatment-related complications (bone marrow, renal, liver, or heart failure, and infections) were included in the malignancy category. Comparison was made between the obese and nonobese patients over the same time period and differences were assessed using chi-square test of significance.

## 3. Results

Of the 849 adult autopsies, 274 cases (32.3%) were obese and 575 (67.7%) were nonobese (Table 1).

In the obese cohort, the BMI ranged from 30 to 80.5 kg/m^2^. Obesity classes I, II, and III accounted for 140 (51%), 66 (24%), and 68 (25%) of the cases, respectively. The patients included 154 males and 120 females with an age range between 18 and 93 years (mean 64) and a weight range from 44 to 255 kg (mean 101).

A review of the patients' medical records revealed a history of DM, HTN, and HL in 35.4%, 60.9%, and 18.2% of obese patients, respectively. In addition to incorporating specific cut-offs for waist circumference, blood pressure (BP), triglyceride (TG), fasting glucose, and high density lipoprotein (HDL), the American Heart Association/National Heart, Lung, and Blood Institute criteria for metabolic syndrome include the use of medication to treat the three latter conditions as well [11]. When all these factors are accounted for, metabolic syndrome was identified in 29% (Table 1).

The leading causes of death in this population were malignancy (86 patients, 31.4%) and infection (71, 25.9%) followed by IHD (35, 12.8%), PE (17, 6.2%), stroke (12, 4.4%), hemorrhage (11, 4%), and non-IHD (11, 4%) (Table 2).

Malignancy accounted for the majority of deaths in the obese (86 patients, 31.4%). The most common category was carcinoma (43 patients, 50%) followed by hematologic malignancy (36 patients, 41.9%) and single cases of splenic angiosarcoma, pancreatic endocrine neoplasm, lung carcinoid, lung hemangioendothelioma, ocular melanoma, pericardial mesothelioma, and uterine malignant mixed mullerian tumor.

The most common carcinomas included those of the pancreas (9 of 86 patients, 10.5%), liver (7 patients, 8.1%), and lung and breast (5 patients each, 5.8%). All pancreatic tumors were invasive ductal adenocarcinomas with the exception of a single case of neuroendocrine carcinoma. All primary liver tumors were hepatocellular carcinomas. Primary lung carcinomas included 3 adenocarcinomas, 1 small cell carcinoma, and 1 sarcomatoid carcinoma (poorly differentiated non-small cell carcinoma with spindle cell features). Invasive ductal carcinomas accounted for 4 of the 5 breast tumors with the remaining tumor being an invasive lobular carcinoma. In the hematologic category, the most common entity was acute myeloid leukemia (AML, 10 patients) followed by multiple myeloma (MM, 6 patients) and diffuse large B cell lymphoma (DLBCL, 5 patients) (Table 3).

Seventy-one individuals (25.9%) died from infections not associated with an underlying neoplastic process or its treatment. The most common infection was pneumonia (42 patients, 59.2%) followed by soft tissue infections including postsurgical, burn-related, and diabetic gangrene (9 patients, 12.7%). Six deaths (8.5%) were secondary to sepsis from gastrointestinal causes (ischemia, perforation, strangulated hernia, and infections). Five patients (7%) died from sepsis secondary to urinary tract infection, 4 (5.6%) from disseminated fungal infections, 3 (4.2%) from infective endocarditis, and 2 (2.8%) from sepsis without an identifiable source.

IHD was the primary cause of death in 35 of the 274 obese individuals (12.8%, 19 males and 16 females). Most patients had HTN (80%), DM (54%), and metabolic syndrome (63%). HL was present in 34%. Of these patients, 69% had previously undergone coronary artery revascularization. Most infarctions involved the left lateral wall of the heart (63%) followed by the posterior wall (40%) and interventricular septum (37%).

**Figure 1:**
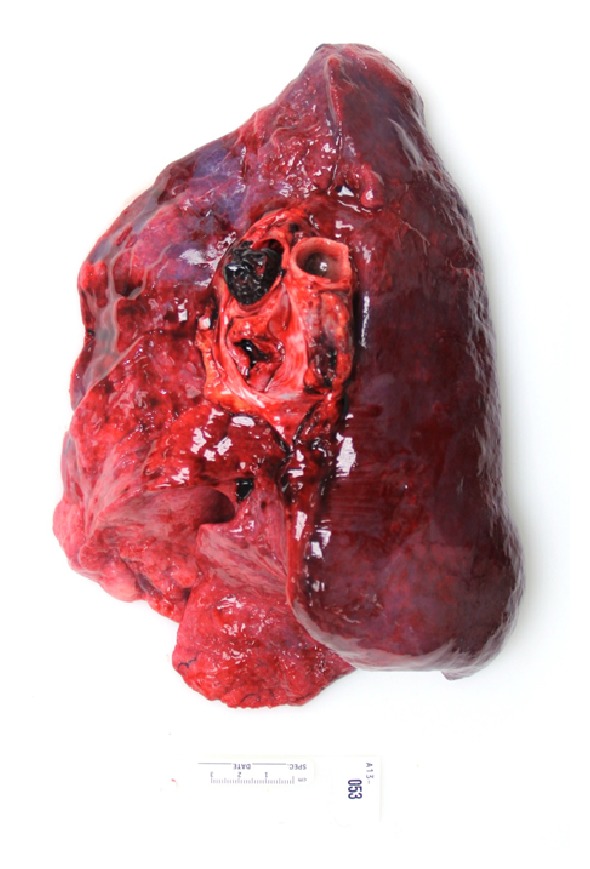
Right lung with a main pulmonary artery embolus.

PE was responsible for the death of 17 obese patients (6.2%, 10 males and 7 females). The mean BMI was 38.9 kg/m^2^. A history of recent major surgery, immobilization, or deep vein thrombosis (DVT) was present in 53%. Autopsy revealed a clinically unsuspected neoplasm in 3 patients: an adrenal cortical adenoma, well-differentiated pancreatic endocrine neoplasm, and metastatic papillary thyroid carcinoma. Clinical suspicion and premortem work-up for PE were initiated in 9 of the 17 individuals (53%). At the time of autopsy, the pulmonary emboli were grossly identified in most cases (82%) and were most commonly located at the bifurcation of the main pulmonary artery (saddle embolus) or in one of its major branches (Figure 1).

Non-IHD was the cause of death in 11 obese patients (4%). Five patients died from congestive heart failure (CHF), 3 from dilated cardiomyopathy (DCM), 2 from cardiac amyloidosis, and 1 from complications of transposition of the great arteries.

Neurological disease accounted for the death of 1 obese patient: an 87-year-old female with a history of dementia for 15 years. Death was attributed to advanced Alzheimer's disease.

**Figure 2:**
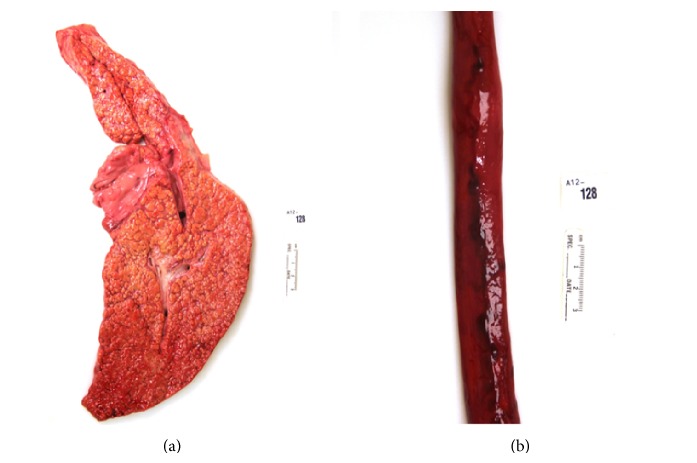
Cirrhosis in the setting of hepatitis C (a). Death resulted from bleeding esophageal varices (b).

Death from liver disease was seen in 8 (2.9%) obese patients. Seven patients died from complications of cirrhosis: 3 were associated with alcohol and 4 with hepatitis C infection and 1 patient died from acute fulminant autoimmune hepatitis (AIH). Complications of cirrhosis included esophageal variceal bleeding (Figure 2), spontaneous bacterial peritonitis, hepatic encephalopathy, and hepatorenal syndrome.

Hemorrhage accounted for 11 deaths (4% of the obese cohort). Five patients died from massive gastrointestinal bleeding (diverticular bleed, perforated duodenal ulcer, gastric ulcers, acute hemorrhagic gastritis, and ischemic colitis), 3 from aortic dissection, 2 from retroperitoneal hemorrhage, and 1 from bilateral adrenal hemorrhage.

The remaining 575 nonobese patients included 310 males and 265 females. Patient age ranged from 18 to 98 years (mean 66). Body weight ranged from 30 to 118 kg (mean 67.5 kg), and BMI ranged from 11.3 to 29.9 kg/m^2^.

A history of DM, HTN, and HL was noted in 17%, 44.5%, and 12.7% of these patients, respectively, and, even in the absence of obesity, 3.3% met the criteria for metabolic syndrome [11] (Table 1).

The leading causes of death in the nonobese were malignancy (187 patients, 32.5%) and infection (137, 23.8%), followed by IHD (10.4%), non-IHD (7.8%), hemorrhage (6.3%), primary diseases of the lung (6.3%), neuromuscular diseases (3%), and PE (3%) (Table 2).

Malignancy also accounted for the majority of deaths in the nonobese population (32.5%). The most common category was carcinoma (55%), followed by hematologic malignancy (40.1%) (Table 3).

Infections not associated with an underlying neoplastic process and represented primarily by pneumonia (66.4%) caused the death of 23.8% of nonobese patients.

Sixty (10.4%) nonobese individuals died from IHD. A history of HTN, DM, and HL was present in 70%, 38%, and 23%, respectively. A history of coronary artery revascularization procedure was present in 38%. Histologic evidence for MI was present in 49 patients (81.7%). The remaining patients had evidence of significant coronary atherosclerosis and cardiac hypertrophy in the setting of exacerbating factors such as sepsis, anemia, hemorrhage, and malignancy.

PE was responsible for the death of 17 nonobese patients (2.9%). A history of recent major surgery, immobilization, or DVT was present in 38% of patients. Four patients had active malignancy at the time of death. There was insufficient time to initiate a work-up for PE in 5 of the 17 patients (collapsed at home or died shortly after arrival to the emergency department). Clinical suspicion and work-up for PE were initiated in 8 of the 12 remaining individuals (67%). Pulmonary emboli were grossly identifiable in most cases (94%) and were most commonly located at the bifurcation of the main pulmonary artery (saddle embolus) or in one of its major branches.

Non-IHD was the cause of death in 45 nonobese patients (7.8%). Twelve deaths were attributed to hypertensive heart disease, 10 to CHF, 8 to valvular disease, 6 to cardiac amyloidosis, and 4 to DCM. The remaining cases included right ventricular dysplasia, sudden cardiac death from left bundle branch block, complications of congenital heart disease, and two cases of arrhythmogenic right ventricular cardiomyopathy.

Neuromuscular disease accounted for 17 deaths (3%) in the nonobese cohort. Eight deaths were secondary to complications of advanced Alzheimer's disease (mean age 79 years). Two patients died from multiple sclerosis. The remaining patients died from a myriad of causes including chronic idiopathic progressive myopathy, amyotrophic lateral sclerosis, central nervous system sarcoidosis with hydrocephalus, multisystem organ failure in the setting of cerebral palsy, and dementia with Lewy bodies.

Based on our findings, obese individuals were statistically more likely to die from PE (6.2% versus 2.9%, *P* = 0.03) and liver diseases (2.9% versus 0.7%, *P* = 0.01) and less likely to die from neurologic diseases (0.4% versus 2.9%, *P* = 0.02) and non-IHD (4% versus 7.8%, *P* = 0.04) (Table 2).

## 4. Discussion

Herein we report a 10-year study of medical autopsy findings in the obese to outline and highlight the differences in the cause of death between this population and nonobese adults. In our study, obese patients accounting for 32% of the total autopsies were more likely to undergo cardiac revascularization procedures and to die from PE and liver disease as compared to nonobese patients.

Obesity in adults has been shown to be associated with a significant reduction in life expectancy for both genders. The Framingham Study found that, at the age of 40 years, the lifespan of individuals with a BMI ≥ 30 kg/m^2^ was on average 6-7 years shorter than those with a BMI ≤ 24.9 kg/m^2^ [15].

Several studies found that certain forms of cancer occur with a greater frequency in the obese population [16–18]. Rapp et al. followed 67,447 men and 78,484 women for approximately 10 years and documented the incidence of cancer in the different BMI categories. In men, there was a significant increase in the incidence of colonic and pancreatic cancer with increasing BMI. In women, the study found a weak positive association between increasing BMI and cancer incidence in general. Women were also significantly more likely to develop non-Hodgkin's lymphomas and uterine cancers [17].

Calle et al. followed more than 900,000 adults in the United States for 16 years and found that obese men and women were more likely to die from cancer than subjects of normal weight. The cancers that occurred with a higher frequency in the obese population included cancers of the esophagus, liver, colorectum, pancreas, gallbladder, kidney, non-Hodgkin lymphoma, and multiple myeloma. Obese men were more likely to die from prostatic and gastric carcinomas and obese women were more likely to die from ovarian, uterine, cervical, and breast carcinomas [19].

In our study, malignancy was the most common cause of death in both obese (31.4%) and nonobese patients (32.5%), a finding that may be partly due to a selection bias in our patient population. The most common type of malignancy in both groups was carcinoma, specifically adenocarcinoma of the pancreas (10.5% obese, 11.2% nonobese), followed by hematologic malignancy (41.9% obese versus 40.6% nonobese) (Table 3). There was no statistically significant difference in any type of malignancy as a cause of death in the two cohorts. However, these findings may not reflect differences in the prevalence of malignancy in the two populations.

Falagas and Kompoti conducted a review of the literature looking for the association of obesity with infection [20]. Obese individuals appeared to have a higher incidence of nosocomial postsurgical infections [21, 22], aspiration pneumonia [23, 24], community acquired pneumonia [25, 26], and skin infections [27, 28]. In our patient population, infection was the second most common cause of death (25.9% obese versus 23.8% nonobese) with pneumonia accounting for 59.2% and 66.4% of the deaths, respectively.

Wilson et al. [29] studied the relationship between being overweight and obese on the development of new cardiovascular disease (CVD) risk factors and vascular disease end points (angina, MI, and CAD). The patient population included Framingham Heart Study participants between the ages of 35 and 75 years, followed up for 44 years. The age-adjusted relative risk (RR) for CVD in obese men and women was 1.46 and 1.64, respectively. Interestingly, the study found that although excess adiposity was associated with a significantly increased RR for cardiovascular events (angina, CAD) in both sexes, only women showed a significantly increased age-adjusted RR for MI and death from CVD (1.68 and 1.67, resp.).

In our study, IHD was the third most common cause of death in both the obese (12.8%) and nonobese (10.4%) populations; however, the difference in the rate of death between the two groups did not reach statistical significance (*P* = 0.341). In addition, there was no significant difference in the extent of significant CAD found at autopsy (45.1% versus 38.8%, *P* = 0.08). However, when the prevalence of IHD/MI was assessed (clinical history and/or postmortem findings), obese individuals were significantly more likely to have had a history of symptomatic IHD/MI (33.6% versus 25.7%, *P* = 0.018) and a history of coronary artery revascularization procedure (20.4% versus 12.5%, *P* = 0.003), possibly reflecting a more vigilant approach from the clinical team.

In this study, obese patients were less likely to die from non-IHD than the nonobese. Eleven obese patients died from non-IHD. Obesity-related cardiomyopathy, a clinical syndrome attributed to cardiac structural and hemodynamic alterations in the setting of obesity [30, 31], was not identified as a cause of death in these patients. However, this could be limited by the lack of specific pathologic findings in this condition. Fryer et al. reviewed 202 medicolegal postmortem autopsies on morbidly obese individuals and identified obesity-related cardiomyopathy as a cause of death in 4 of 6 possible cases [32]. Obesity-related cardiomyopathy remains a difficult diagnosis for the pathologist to make. It requires careful clinical-pathologic correlation and the exclusion of other significant findings that could be implicated in mortality.

Obesity has been shown to be a risk factor for the development of DVT [33] and venous thromboembolic events (VTE) [34]. Ageno et al. conducted a meta-analysis to evaluate the prevalence of major cardiovascular risk factors in patients with VTE compared to control subjects. The meta-analysis included 21 case-control and cohort studies and a total of 63,552 patients. When compared to control subjects, obese individuals had an odds ratio (OR) of 2.33 for developing VTE [35]. Stein et al. utilized the National Hospital Discharge Survey database to investigate the risk of obesity on VTE. Compared to nonobese individuals, the RR of DVT was 2.50 and that of PE was 2.21. Moreover, obesity had the greatest impact on people younger than 40 years [36]. Our study similarly showed that obese individuals were significantly more likely to die from PE than nonobese.

Obese individuals were more likely to die from liver disease and complications of cirrhosis. Of the 8 obese patients that died from liver disease, several had DM, HL, and a low HDL level. Patients with nonalcoholic fatty liver disease (NAFLD) are more likely to experience disease progression in the setting of BMI ≥ 28 kg/m^2^ [37], DM [38], and higher visceral adiposity index [39]. Such risk factors may have played a role in the progression of the primary liver disease in our patients.

Several disease entities occur more frequently in the obese and many are clinically unsuspected or undiagnosed. Gabriel et al. retrospectively reviewed the autopsies of 311 patients, 125 of whom were obese. The authors found that this population is 1.65 times more likely to have major clinically significant unsuspected diagnoses on postmortem examination compared with underweight and normal weight populations [40]. Specifically, PE was the most frequently missed clinically significant diagnosis. Had these diagnoses been suspected, they might have led to a change in management and possibly prolonged the life of the patient.

We aimed at determining and comparing the cause of death in a hospital-based population of obese and nonobese patients. Our study is limited by the fact that only a fraction of the population that died in our hospital was consented for postmortem examination. Moreover, because the primary objective was to evaluate the cause of death, any comorbid condition or autopsy finding, be it related or unrelated to obesity, was not fully addressed. For instance, although death from different malignancies was not significantly different in the two cohorts, the same may not hold for the prevalence of malignancy in these patients. Similarly, although a significant difference in IHD as a cause of death was not identified, its prevalence was significantly higher in the obese cohort.

Obesity is becoming a major public health concern and the number of postmortem examinations being performed on obese individuals is on the rise [41, 42]. Autopsies on obese individuals constitute one-third of all adult medical autopsies in our center and are technically more challenging, performed with the use of a bariatric autopsy table. While the exact role of obesity in causing these deaths is likely multifactorial and will require further investigation, the significant role of obesity and obesity-related diseases in ultimate death should not be overlooked as several causes of death are shown to differ in this population compared with nonobese adults, such as an increased rate of death from PE and liver disease. It is therefore essential that clinicians develop a heightened awareness of PE and chronic liver disease in this population and institute prophylactic measures when indicated. It is also the pathologist's role to recognize these findings on postmortem examination, if present. Aggressive surveillance, prevention, and treatment in our center likely account for the notable finding that, compared to the nonobese, obese individuals are not more likely to die from IHD. Ultimately, autopsy findings in obese individuals should contribute to overall premortem disease detection, prevention, and management in this population.

## Figures and Tables

**Table 1:** Characteristics of the obese and nonobese patient populations.

849 adult autopsies		
	Obese	Nonobese
*N* (%)	274 (32.3)	575 (67.7)
M : F	154 : 120 (1.28)	310 : 265 (1.17)
Age range-years (mean)	18–93 (64)	18–98 (66)
BMI kg/m^2^ range	30–80.5	11.3–29.9
Obesity class I (BMI 30–34.9)	140 (51%)	—
Class II (BMI 35–39.9)	66 (24%)	—
Class III (BMI ≥40)	68 (25%)	—
Hypertension	167 (60.9%)	256 (44.5%)
Diabetes	97 (35.4%)	98 (17%)
Elevated triglyceride	50 (18.2%)	73 (12.7%)
Metabolic syndrome^*^	80 (29%)	19 (3.3%)

^*^American Heart Association/National Heart, Lung, and Blood Institute criteria (any 3 of 5 constitute diagnosis of metabolic syndrome): (1) elevated BP: ≥130 mm Hg systolic or ≥85 mm Hg diastolic or on antihypertensive drug treatment in a patient with a history of HTN, (2) elevated TG ≥150 mg/dL or on drug treatment for elevated TG, (3) fasting glucose ≥100 mg/dL or on drug treatment for elevated glucose, (4) elevated waist circumference: men ≥102 cm, women ≥88 cm, and (5) reduced HDL-C <40 mg/dL in men or <50 mg/dL in women or on drug treatment for reduced HDL-C [11].

**Table 2:** Comparison of the cause of death in obese and nonobese individuals.

Cause of death	Obese (274)		Nonobese (575)		*P* value
Malignancy	86	31.4%	187	32.5%	**0.785**
Infection	71	25.9%	137	23.8%	**0.566**
Ischemic heart disease	35	12.8%	60	10.4%	**0.341**
Stroke	12	4.4%	15	2.6%	**0.176**
Pulmonary embolism	17	6.2%	17	2.9%	**0.027**
Hemorrhage	11	4.0%	36	6.3%	**0.193**
Genitourinary	5	1.8%	7	1.2%	**0.486**
Liver	8	2.9%	4	0.7%	**0.011**
Lung	10	3.6%	36	6.3%	**0.126**
Skin	1	0.4%	1	0.2%	**0.592**
Nonischemic heart disease	11	4.0%	45	7.8%	**0.043**
Neurologic	1	0.4%	17	3.0%	**0.015**
Gastrointestinal ischemia	4	1.4%	5	1.0%	**0.434**
Hematologic	2	0.8%	0	0%	**0.040**
Undetermined	0	0%	8	1.4%	**0.051**

**Table 3:** Malignancy in different organ systems as a cause of death in obese and nonobese individuals.

Malignancy	Obese (86)		Nonobese (187)		*P* value
Hematologic^*^	36	41.9%	76	40.6%	**0.849**
Carcinoma^†^					
Pancreas	9	10.5%	21	11.2%	**0.851**
Lung	7	8.1%	25	13.4%	**0.212**
Colon	0	0%	8	4.3%	**0.052**
Liver	7	8.1%	7	3.7%	**0.126**
Breast	5	5.8%	5	2.7%	**0.199**
Biliary	4	4.7%	6	3.2%	**0.555**
Kidney	1	1%	5	2.7%	**0.429**

^*^Includes lymphomas of different organ systems.

^†^Includes the organs that were most commonly involved by carcinoma and is not an exhaustive list of all tumors identified.
